# Bilateral widespread segmental swelling on nerve sonography in multifocal acquired demyelinating sensory and motor neuropathy: Two case reports

**DOI:** 10.1097/MD.0000000000027900

**Published:** 2021-11-19

**Authors:** Wan-Jen Hsieh, Kai-Chieh Chang, Hsueh-Wen Hsueh, Chi-Chao Chao, Sung-Tsang Hsieh

**Affiliations:** a Department of Neurology, National Taiwan University Hospital, College of Medicine, National Taiwan University, Taipei, Taiwan; b Department of Neurology, National Taiwan University Hospital Yunlin Branch, College of Medicine, National Taiwan University, Yunlin, Taiwan.

**Keywords:** bilateral swelling, case report, multifocal acquired demyelinating sensory and motor neuropathy, nerve ultrasound

## Abstract

**Introduction:**

Multifocal acquired demyelinating sensory and motor neuropathy (MADSAM) is an asymmetric immune-related neuropathy with conduction block. We report 2 MADSAM cases with detailed clinical, electrophysiological, and sonography profiles.

**Patient concerns and diagnosis:**

Two cases presented with patchy sensorimotor impairment in both clinical and electrophysiological findings. Notably, nerve ultrasound demonstrated multifocal nerve enlargement not only at sites of conduction blockade but also at the unaffected contralateral sites. Interestingly, in our first case, focal radial nerve enlargement was observed prior to the clinical manifestations, suggesting nerve dynamic pathogenesis with variable clinical significance.

**Interventions and outcomes:**

The first patient was initially treated with prednisolone, however, 3 months after steroid therapy, her symptoms progressed. After treatment with intravenous immunoglobulin for 3 months, the symptoms stabilized. The second patient showed improvement after 2 months of prednisolone treatment.

**Conclusion:**

These observations suggest a more widespread pathomechanism underlying MADSAM, and ultrasound may detect nerve lesions earlier than clinical electrophysiology studies, and is warranted for early detection and thorough documentation of nerve pathology.

## 1 Introduction

Multifocal acquired demyelinating sensory and motor neuropathy (MADSAM), a variant of chronic inflammatory demyelinating polyneuropathy (CIDP), is characterized by chronic asymmetric sensorimotor involvement with conduction blocks.^[1]^ Ultrasound has been used widely as an imaging tool to evaluate different neuropathies.^[2]^ Although numerous nerve ultrasound studies in classical CIDP have been published, MADSAM ultrasound studies are still scarce.^[3–8]^ CIDP and MADSAM have been observed to share similar heterogeneous echointensity patterns (hypoechogenicity or mixed echogenicity) in enlarged nerves, but the nerve swelling pattern differs between these 2 diseases: diffuse enlargement with a varying predominance in CIDP versus regional nerve enlargements corresponding to conduction blocks in MADSAM.^[2]^ As bilateral sampling is performed routinely in nerve conduction studies (NCSs), whether bilateral sampling is recommended in sonography remains undetermined. In this study, we described 2 patients with MADSAM presenting bilateral widespread segmental swelling on nerve sonography and further reviewed 8 other published MADSAM cases with nerve ultrasound data.

## 2 Case presentation

### 2.1 Case 1

A 43-year-old woman presented with an 8-year history of asymmetric peripheral neuropathy. Initially, the patient developed numbness in her right toes in 2012 with ptosis of the right eye. The ptosis was pupil-sparing and without diurnal changes. In 2014, the numbness progressed to her right ankle. In 2017, right wrist drop gradually developed, which was accompanied by numbness in her right 1st to 3rd fingers. Furthermore, she experienced difficulty when opening bottles and holding chopsticks with her right hand. In 2020, left foot numbness followed by numbness in her left 1st to 3rd fingers gradually developed. Neurological examinations revealed weakness in right thumb adduction and opposition and in right wrist extension. Generalized hyporeflexia with an absent deep tendon reflex was recorded in the right upper limb. Sensory examination revealed a symmetrical decrease in vibration in both thumbs and large toes.

**Figure 1:**
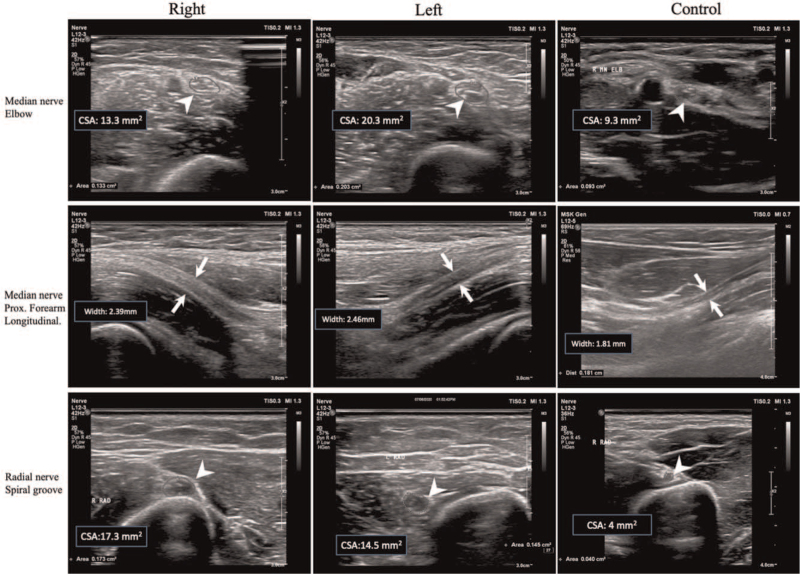
Bilateral swelling in the bilateral median and radial nerves in case 1. Ultrasonography showing enlargement of the bilateral median nerves in the elbow region and the bilateral radial nerves in the spiral groove region. Swelling was not observed in the control. CSA = cross-sectional area.

An NCS revealed conduction blocks in the bilateral median nerves of the forearms and in the right radial nerve across the spiral groove. The F wave was absent in the bilateral median nerves, and the minimal latency in the bilateral tibial nerves was prolonged (see Table S1, supplementary digital content, which illustrates the conduction blocks in the NCS). Routine blood tests on admission and a further workup of secondary causes of asymmetric peripheral neuropathy revealed no specific abnormalities except for the cerebrospinal fluid study, which revealed increased total proteins (66.4 mg/dL) and a normal cell count and immunoglobulin G index (0.6). MADSAM was diagnosed based on the clinical presentation and NCSs. Notably, nerve sonography revealed segmental swelling at the bilateral median and bilateral radial nerves and right cervical spinal nerve even in segments not involved in neurological examinations or NCSs (Fig. 1).

The patient was treated with 3 days of methylprednisolone (500 mg/d), followed by an oral taper with prednisolone. However, 3 months after steroid therapy, her symptoms progressed. The numbness of her left hand extended to the palm, and her left wrist weakness deteriorated. Therefore, she was treated with intravenous immunoglobulin (2 g/kg) for 5 days every 3 months. Her disease then stabilized.

### 2.2 Case 2

An 18-year-old man without systemic disease presented with acute-onset progressive blurring in the right eye, diplopia, and ptosis for 5 days. His right ocular motility was almost completely restricted, except for lateral gaze. Furthermore, his right pupil was involved. Two weeks before admission, numbness in his left big toe presented and gradually spread to the entire left sole. He denied recent illnesses or vaccination. According to the patient, he had 2 episodes of left abducens nerve palsy and distal hand numbness over the past 4 years. Each time, after receiving oral prednisolone 15 mg for 1 week, the symptoms resolved.

**Figure 2:**
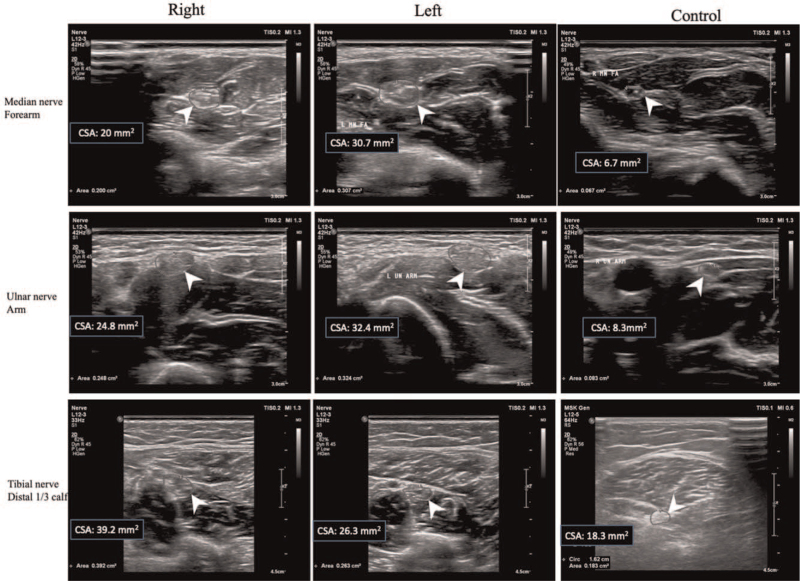
Bilateral swelling in the bilateral median, ulnar, and tibial nerves in case 2. Ultrasonography showing enlargement of the bilateral median nerves in the forearm regions, the bilateral ulnar nerve in the arm region, and the bilateral tibial nerve at the distal third leg, with an increased cross-sectional area (CSA). Swelling was not observed in the control.

Neurological examination revealed complete right oculomotor palsy. His right eye was in a down and out position. Ocular motility examination revealed a complete deficit in adduction, supraduction, and infraduction for the right eye. His right pupil was 7 mm in size as opposed to 4 mm on the left. His right eye was also not reactive to light, and no relative afferent pupillary defect was identified. Furthermore, he also showed weakness in left ankle dorsiflexion and generalized hyporeflexia. Sensory examination revealed decreased pin-prick and light touch sensations over his left sole, big toe, and second toe. NCSs showed conduction blocks in the left median, left peroneal, and left tibial nerves and slow conduction velocities in the bilateral median and left tibial nerves. F waves in the bilateral tibial, left median, left ulnar, and left peroneal nerves were absent (see Table S1, supplementary digital content, which illustrates the conduction blocks in the nerve conduction study). Cerebrospinal fluid analysis revealed cytoalbuminologic dissociation, with a total protein concentration of 50.8 mg/dL. Thyroid function, VitB12, folic acid, hepatitis C virus, hepatitis B virus, human immunodeficiency virus, cryoglobulin, electrophoresis, and autoimmune profiles such as antinuclear antibody, anti-extractable nuclear antigen, antineutrophil cytoplasmic antibody, anti-sjögren's-syndrome-related antigen A, anti-sjögren's-syndrome-related antigen B, anti-dsDNA, dilute russell viper venom test, anti-cardiolipin, and anti-B2 GP1 were assessed, which were unremarkable. MADSAM involving cranial nerves and nerves in the limbs was diagnosed. Neuromuscular sonography revealed segmental swelling at the bilateral median, bilateral ulnar, bilateral tibial, and right sural nerves (Fig. 2). The patient was treated with steroid therapy with 500 mg methylprednisolone per day for 3 days, followed by an oral taper with prednisolone. Two months after steroid therapy, his symptoms showed marked improvement except for a sluggish light reflex in his right eye. Moreover, follow-up sonography revealed reduced swelling in some previously involved nerves (see Table S2, supplementary digital content, which illustrates the cross-sectional area change on sonography).

## 3 Discussion

Our study presents detailed clinical profiles, electrophysiological study results, and nerve sonography data for 2 patients with MADSAM. Two important findings were uncovered: bilateral widespread nerve swelling on sonography with asymmetric clinical and electrophysiological findings and nerve swelling 3 months prior to clinical weakness development.

In the approach for neuropathy, a nerve conduction study in bilateral nerves is suggested. However, whether bilateral sampling should be recommended in sonography remains undetermined. Our case reports revealed multifocal nerve enlargement not only at sites of conduction blockade but also at the unaffected contralateral sites. To confirm these findings, we reviewed 8 other MADSAM cases with nerve ultrasound studies published from 2012 to 2020 (Table 1).^[3–8]^ Nerve enlargement was found at the sites of conduction blocks in all cases. In the report for case 7, whether left-side nerve sonography was performed was not reported. After excluding this case, 7 other cases (cases 3, 4, 5, 6, 8, 9, and 10, Table 1) also reported bilateral swelling in some of the nerves studied by sonography. Thus, we recommend bilateral sampling in sonography.

**Table 1:** Clinical, electrophysiological, and sonographic studies of patients with MADSAM in our study and the literature.

	Clinical presentation			
Patient/age/sex (source)	Weakness	Numbness	Nerve conduction study	Enlargement in sonography
1/43/F [Case 1]	Rt. ptosis and Rt. radial and median ns. -Lt. wrist drop several months after the echo	Bilateral fingers and Lt. foot	CBs at the bilateral median, tibial, Rt. radial, and peroneal ns.	Bilateral median n. from the elbow to the forearm; bilateral radial ns. at the spinal groove, right C7 cervical spinal ns.
2/18/M [Case 2]	Rt. CN3 palsy Lt. peroneal n.	Lt. sole, big toe, and second toe	CBs at the Lt. median, peroneal, and tibial ns.	Bilateral median n. from the elbow to the forearm; bilateral ulnar n., particularly the upper arm; Rt. sural n. at the ankle level
3/41/M ^[7]^	Lt. ulnar and peroneal ns.	Lt. ulnar and peroneal ns.	CBs at the Lt. common peroneal n. at the fibular head and Lt. ulnar n. in the forearm	Lt. radial n., spiral groove; Lt. median n., upper arm; Lt. ulnar n., condylar groove and the distal forearm; Lt. peroneal n., distal part of the popliteal fossa; bilateral median nerves, forearm
4/51/F ^[7]^	Partial Rt. median n. and Lt. ulnar and peroneal ns.	Lt ulnar and peroneal ns.	CBs at the mid-upper arm of the Lt. ulnar n.	Lt. ulnar n.; Lt. peroneal n., proximal part of the popliteal fossa; mild swelling in the bilateral median n., upper arm.
5/18/M ^[6]^	Rt. facial n. Rt. hand	Rt. hand	CBs at the Rt. median and Lt. ulnar ns. between the elbow and axilla	Bilateral median nerve, axilla; Lt. ulnar nerve, elbow; Lt. vagus nerve, above the br. point of the LRN; Rt. facial nerve under the parotid gland
6/51/M ^[3]^	Intermittent fine motor dysfunction in the Lt. hand (digits I–III)	Lt. hand Rt. toe, forefoot, and sole	CBs at the Rt. peroneal and Lt. median ns.	Lt. median n. at the wrist, elbow and upper arm; bilateral tibial n. at the distal part; Rt. tibial n. at the proximal part
7/61/M ^[5]^	Rt. lower leg, particularly plantar flexion	Rt. plantar ascending paresthesia	CBs at the Rt. tibial nerve at the level of the popliteal fossa	Rt. tibial nerve in the proximal part of the popliteal fossa
8/71/F ^[8]^	Bilateral hands	Bilateral hands	CBs at the Rt. median n. in the forearm (cubital fossa) -Decreased velocity in the bilateral median and ulnar ns.	Rt. median n. in the cubital fossa; bilateral proximal median and ulnar ns., Lt. >Rt. brachial plexus and spiral n.
9/57/M ^[8]^	Rt. intrinsic hand muscles (more severe in the median n. groups)	Rt. ulnar distribution	CB at the Rt. median n. in the forearm -Decreased SNAP in the Rt. median and ulnar ns.	Rt. median nerve at the wrist Asymmetric brachial plexus, marked on the right
10/middle aged/unknown ^[4]^	Asymmetric tetraparesis -Dysphonia and dysphagia	Asymmetric numbness in the 4 limbs	CBs at the median and ulnar ns. and the Lt. tibial n. -Absent SNAP in the median and ulnar ns.	Bilateral median n.; Rt. vagus n.; Lt. peroneal n.; Rt. proximal tibial n.

br. = branching, CB = conduction block, LRN = left recurrent nerve, Lt. = left, n. = nerve; ns. = nerves, Rt. = right.

The second important finding is that nerve swelling may be evident several months prior to clinical symptom development. Our first case showed no response to initial steroid therapy, which allowed us to observe the patient's subsequent weakness and numbness several months after sonography. Among the other 8 cases, 4 (cases 3, 4, 5, and 6) showed sonographic swelling in areas that were clinically and electrographically unaffected (Table 1), but whether symptoms subsequently developed was not reported. Combining these 2 findings, we hypothesized that a pathomechanism (e.g., blood-nerve barrier breakdown)^[1]^ already subclinically affected bilateral nerves and led to nerve swelling before clinical and electrophysiological abnormalities appeared. The asymmetry of the clinical and electrophysiological patterns suggested a breakthrough of nerve function disturbance by this pathomechanism. This hypothesis may explain that a part of patients with MADSAM would evolve into a typical CIDP.^[9]^

## 4 Remarks

In summary, our report indicated 2 important findings: bilateral widespread nerve swelling on sonography with asymmetric clinical and electrophysiological findings and nerve swelling 3 months prior to the development of clinical symptoms. These findings may suggest a bilateral widespread pathomechanism in MADSAM, and ultrasonography might detect nerve lesions before clinical symptoms and abnormalities in electrophysiological studies appear.

## Acknowledgments

This study was initiated by the principal investigator and conducted without external funding. The authors confirm that we have read the Journal's position on issues involved in ethical publication and affirm that this report is consistent with those guidelines.

## Author contributions

All authors contributed to data acquisition and analysis. Wan-Jen Hsieh and Hsueh Wen Hsueh wrote the manuscript with input from all authors.

**Conceptualization:** Wan-Jen Hsieh, Kai-Chieh Chang, Hsueh Wen Hsueh, Chi-Chao Chao, Sung-Tsang Hsieh.

**Data curation:** Kai-Chieh Chang, Hsueh Wen Hsueh, Chi-Chao Chao, Sung-Tsang Hsieh.

**Supervision:** Chi-Chao Chao, Sung-Tsang Hsieh.

**Writing – original draft:** Wan-Jen Hsieh.

**Writing – review & editing:** Hsueh Wen Hsueh, Chi-Chao Chao, Sung-Tsang Hsieh.

## Supplementary Material

Supplemental Digital Content
