# Supplementary material for: Bilateral widespread segmental swelling on nerve sonography in multifocal acquired demyelinating sensory and motor neuropathy: Two case reports
Source: Medicine (Baltimore). 2021 Nov 19;100(46):e27900. doi: 10.1097/MD.0000000000027900 (PMC8601274; doi:10.1097/MD.0000000000027900)
Supplement: Supplemental Digital Content [file medi-100-e27900-s001.doc]

**Legends: The nerve conduction study revealed multiple conduction block in both cases.**

**Table S1.** Conduction block in the nerve conduction study

| Nerve | Latency (ms) | Amplitude (mv) | Motor NCV (m/s) |
| --- | --- | --- | --- |
| **Case 1 pretreatment** | | | |
| ***Median R*** |  |  |  |
| Wrist | 3.9 | 8.8 |  |
| Elbow | 7.2 | 5.0 | 52 |
| ***Median L*** |  |  |  |
| Wrist | 4.0 | 7.5 |  |
| Elbow | 7.6 | **3.2** | 52 |
| ***Radial R*** |  |  |  |
| Forearm | 2.2 | 9.6 |  |
| Elbow | 4.4 | 9.6 | 54 |
| Spiral groove | 6.4 | **2.0** | 51 |
| ***Radial L*** |  |  |  |
| Forearm | 1.5 | 7.0 |  |
| Elbow | 3.4 | 6.8 | 57 |
| Spiral groove | 5.7 | 5.2 | 59 |
| **Case 2 pretreatment** | | | |
| ***Median R*** |  |  |  |
| Wrist | 3.9 | 12.6 |  |
| Elbow | 8.4 | 11.0 | **47** |
| ***Median L*** |  |  |  |
| Wrist | 2.8 | 9.8 |  |
| Elbow | 8.1 | **3.7** | **42** |
| ***Tibial L*** |  |  |  |
| Ankle | 3.2 | 15.8 |  |
| Popliteal fossa | 13.4 | **5.1** | **36** |
| ***Peroneal L*** |  |  |  |
| Ankle | 3.8 | 4.6 |  |
| Bl fibula | 9.5 | **1.2** | 47 |
| Knee | 11.1 | **1.1** | 50 |

NCV, nerve conduction velocity

**Legends: The nerve ultrasound revealed decreased swelling size in the Case 2.**

**Table S2.** Nerve ultrasound results in case 2

|  | Pretreatment CSA (mm^2^) | Posttreatment CSA (mm^2^) | Norm^1^ |
| --- | --- | --- | --- |
| **Right median nerve** |  |  |  |
| Wrist | 10.7 | 10.7 | 10.07 ± 2.27 |
| Forearm | **20** | **21** | 8.13 ± 2.6 |
| Elbow | **20.8** | **12.7** | 9.96 ± 2.47 |
| **Left median nerve** |  |  |  |
| Wrist | 9.7 | 10.2 | 10.07 ± 2.27 |
| Forearm | **30.7** | **21.7** | 8.13 ± 2.6 |
| Elbow | **21.2** | **13.3** | 9.96 ± 2.47 |
| **Right radial nerve** | **7.8** | 6.2 | 4.71 ± 1.15 |
| **Left radial nerve** | **7** | 4.8 | 4.71 ± 1.15 |
| **Right ulnar nerve** |  |  |  |
| Wrist | 5.5 | 5.7 | 5.52 ± 1.41 |
| Elbow | 9.3 | 7.0 | 8.75 ± 3.2 |
| Arm | **24.8** | 22.6 | 7.43 ± 3.0 |
| **Left ulnar nerve** |  |  |  |
| Wrist | 7.7 | 8.2 | 5.52 ± 1.41 |
| Elbow | **11.1** | 9.5 | 8.75 ± 3.2 |
| Arm | **32.4** | **32.4** | 7.43 ± 3.0 |
| **Right tibial nerve** |  |  |  |
| Tibial nerve | 15.4 | 12 | 14.5 ± 5.35 |
| 1/3 tibial | **39.2** | 16 | -^2^ |
| **Left tibial nerve** |  |  |  |
| Tibial nerve | 16.2 | 21.4 | 14.5 ± 5.35 |
| 1/3 tibial | **26.3** | 25.2 | -^2^ |
| **Right sural nerve** | **9.4** | 4.5 | 3.5 ± 1.16 |
| **Left sural nerve** | **7.7** | - | 3.5 ± 1.16 |

CSA, cross-sectional area.

^1^: According to our own published data, HW Hsueh et al., Front Neurol. 2020.

^2^: No available data but was considered similar to the segment at the ankle.
